# Biodegradable Dissolved Organic Carbon (BDOC) Removal from Micro-Polluted Water Source Using Ultrafiltration: Comparison with Conventional Processes, Operation Conditions and Membrane Fouling Control

**DOI:** 10.3390/polym14214689

**Published:** 2022-11-03

**Authors:** Ming Chen, Shuhuai Shen, Fan Zhang, Cong Zhang, Jianglei Xiong

**Affiliations:** 1 School of Civil Engineering, Southeast University, Nanjing 210096, China; 2 Huzhou Ecological Environment Bureau, Changxing Branch, Huzhou 313100, China; 3 China Electronics System Engineering No.2 Construction Co., Ltd., Wuxi 214115, China

**Keywords:** ultrafiltration, drinking water, biological stability, BDOC, membrane fouling

## Abstract

The biodegradable dissolved organic carbon (BDOC) in micro-polluted water sources affects the drinking water quality and safety in the urban water supply. The conventional technology of “coagulation-sedimentation-filtration” in a water plant located in the lower reaches of the Yangtze River removed dissolved organic carbon (DOC) with a molecular weight (MW) > 30 kDa effectively, but the BDOC elimination only ranged 27.4–58.1%, due to their predominant smaller MW (<1 kDa), leading to a high residual BDOC of 0.22–0.33 mg/L. To ensure the biological stability of drinking water, i.e., the inability to support microbial growth (BDOC < 0.2 mg/L), a pilot-scale ultrafiltration process (UF, made of aromatic polyamide with MW cut-off of 1 kDa) was operated to remove BDOC as an advanced treatment after sand-filtration. Results showed the membrane flux decreased with the increase in the influent BDOC concentration and decrease in operating pressure. With an operating pressure of 0.25 MPa, the BDOC removal by UF reached 80.7%, leading to a biologically stable BDOC concentration of 0.08 mg/L. The fouling of the membrane was mainly caused by organic pollution. The H_2_O_2_–HCl immersion washing method effectively cleaned the membrane surface fouling, with a recovery of membrane flux of 98%.

## 1. Introduction

The occurrence of biodegradable dissolved organic carbon (BDOC) and assimilable organic carbon (AOC) in drinking water produced by waterworks provides potential nutrient substrates in the water distribution system for the re-growth and reproduction of heterotrophic bacteria that have not been killed when not enough chlorine is left in the pipes [1,2,3,4]. Bacteria can use the BDOC and AOC in the water as a nutrient matrix to re-grow, threatening drinking water safety, and attach to the water supply pipe’s inner surface to form biofilms, causing corrosion and scaling of the pipe [5,6]. The existence of a biofilm is further conducive to the growth and spread of pathogenic microorganisms in the water of the pipe network, posing a threat to public health [7]. However, increasing the amount of chlorine after sand filtration causes disinfection byproducts. In addition, the scaling and corrosion of the pipe wall reduce the water-delivery capacity and increase the energy consumption of the secondary pumping station [8]. The biostability of drinking water refers to the potential of BDOC and AOC in drinking water to support the growth of heterotrophic bacteria [9,10,11]. The high biostability of drinking water illustrates that the content of organic nutrients required for bacterial growth in water is low, and bacteria do not grow easily; in contrast, bacteria breed easily. Only by controlling BDOC and AOC in the treated water can the problem of bacterial re-growth in the pipe network be fundamentally solved, especially BDOC, because the concentration of AOC is usually much lower than BDOC in water [4]. Generally, when the BDOC in the water body is less than 0.2 mg/L, the water quality can reach biological stability [12]. The guarantee of biological stability in water by conventional treatment processes is usually unstable and greatly affected by water quality, water temperature, and treatment methods. Generally, conventional treatment processes have limited ability to remove organic matter with small molecular weights, including BDOC in water [13].

Ultrafiltration (UF) separation is a promising purification strategy for advanced water and wastewater treatment. As a semipermeable membrane, UF membranes can be used to separate solutes and colloidal particles (e.g., proteins, bacteria, viruses, and organics) from those with a very low molecular weight (including the water molecules) by applying a pressure or concentration gradient on both sides of the membrane [14,15,16]. Ultrafiltration has been utilized widely in wastewater treatment and water reclamation due to the advantages of low energy consumption and high separation efficiency [17,18]. Ultrafiltration has a good effect on removing particles, colloids, and bacteria in water, but it can hardly retain inorganic ions [19,20,21]. At present, organic ultrafiltration membrane materials mainly include acetate fiber (CA), polyvinyl chloride (PVC), polysulfone (PS), polyamine (PA or nylon), and polyvinylidene fluoride (PVDF) [22,23,24]. Membranes made with different polymeric materials show various advantages and are applied in varied scenarios, due to their varied mechanical, chemical, and hydrophilic or hydrophobic properties. The performance of UF (e.g., flux, contaminant removal, and fouling) can be affected by various factors, including temperature, operating pressure, influent concentration, liquid properties, and running time. Under normal operation, the pressure determines the membrane flux, removal rate, and operating cost, so it directly determines the operating performance and economy of the UF technology. The influent concentration directly affects the fouling of the membrane. An excessive influent concentration can easily cause membrane blockage and affect the filtration and operating life of the membrane. After a long-duration filtration process, the substances in the inflow gradually attach and compact on the membrane surface [25]. Membrane fouling means that the particles, colloids, or solute macromolecules in the treated solution form an adhesion layer on the membrane surface or adsorb and deposit in membrane pores due to physical, chemical, or mechanical interactions with the membrane, causing the pores to be blocked [26]. The solute blockage causes the filtration resistance to increase, and the gel layer formed on the membrane surface increases the mass transfer resistance. Membrane fouling is a dynamic process, depending on the physical and chemical characteristics of the membrane, the characteristics of the feed and liquid, and the operating conditions of the UF [27]. Reversible fouling can be eliminated by periodic backwashing, while irreversible fouling of membranes must apply combined chemical cleaning and certain hydraulic flushing to restore the UF membrane performance.

The organic matter contained in the water, especially BDOC, is the organic nutrient matrix for the growth of bacteria in the water supply system. This study investigated the molecular weight distribution of organics in the water resource of the Yangtze River, and their removal during varied treatment processes in the conventional waterworks. According to the molecular weight distribution of organics, and to enhance BDOC removal based on the existing processes in the water plant, a pilot-scale UF process (made of aromatic polyamide with MW cut-off of 1000 Da) was applied and studied for water purification after sand filtration. Because of the small pore size, microorganisms could also be rejected by the UF membrane. The operating conditions, including pressure, influent concentration, and running cycle, were focused. The recovery of membrane flux by cleaning it after fouling was studied. The research objectives were to verify the feasibility of UF to improve the biological stability of water during water treatment, and to optimize UF operation. Investigating conventional treatment processes and UF separation could facilitate the selection of proper methods to enhance the biological stability of water. 

## 2. Materials and Methods

### 2.1. Materials

All reagents were prepared using analytical-grade chemicals, used as-received without further purification. Hydrochloric acid (HCl, 37%), sodium hydroxide (NaOH, 99%), and hydrogen peroxide (H_2_O_2_, 3%) were supplied by the Sinopharm group Co. Ltd. (Beijing, China), used for cleaning UF membranes after preparing diluted solutions. Ultrapure water (18.2 MΩ/cm, Milli-Q system) was used as the reagent water and for cleaning membranes in experiments. The real water samples were taken from the effluent of a sand filter of a water treatment plant located in Nanjing, China, where Yangtze River water was used as the drinking water source. The qualities of water samples for pilot-scale UF experiments are shown in Table S1 in the Supplementary Materials.

### 2.2. Pilot-Scale UF Process Setup

**Figure 1:**
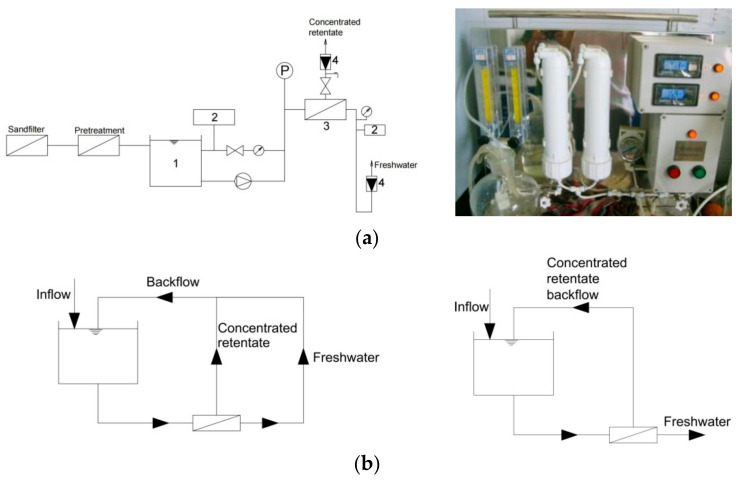
The scheme of pilot-scale UF process. (**a**) UF process and the photo of UF modules; 1: Tank, 2: Conductivity meter, 3: UF module, 4: Flowmeter. (**b**) Varied operating methods.

The pilot-scale UF process is shown in Figure 1a. The raw water after sand filtration was pumped into a dead-end micro-filter with a pore size of 0.45 µm to sieve large particles and reduce the burden of UF membranes. Then, the pretreated water in the tank was filtered using two UF modules (from Sepro, Carlsbad, CA, USA). The UF module was made of an aromatic polyamide roll membrane with an MW cut-off of 1000 Da, and a membrane surface area of 0.8 m^2^. The membranes made of polyamide (or nylon) generally have merits of high hydrophilic nature, mechanical and chemical stability, and durability. The membrane operating pressure and flux ranged from 0.1–0.5 MPa and 10–45 L/(m^2^·h), correspondingly. The UF process was operated in two different ways as shown in Figure 1b based on the experimental design. The retentate and filtrate both flew back to the tank during one operation cycle to keep the composition of the raw water constant. No obvious variation of BDOC in the feed water was observed. On the other hand, only retentate flew back to the tank to increase the concentration of influent gradually. After each experiment, the membrane module was cleaned with ultrapure water for 30 min with pressures ranging from 0.3 to 0.5 MPa.

### 2.3. BDOC Removal by UF

The key parameters of the UF process are the operating pressure (*P*), influent concentration (*C*), and running cycle or time (*t*), and the effects on the BDOC removal and membrane flux of raw water were analyzed. The operating pressure was adjusted in the range of 0.1–0.5 MPa, and both the retained concentrated solution and permeate freshwater flew back to the tank (Figure 1b), keeping a constant concentration of inflow, to investigate the effect of operating pressure. Samples were taken at different time intervals to determine the contaminants’ removal. To investigate the effect of the influent concentration, the concentrated solution returned back to the tank (Figure 1b), so the influent concentration of BDOC increased from 0.3 to 6.53 mg/L with the operating time. The pressures were recorded using a pressure gauge. The membrane flux (*J*), BDOC removal (*R*), and freshwater yield (*Y*) were calculated as shown in Equations (1)–(3).

(1) $$J=\frac{Q}{A}$$

(2) $$R=\left(1-\frac{C_{e}}{C_{i}}\right)\times 100\%$$

(3) $$Y=\frac{Q}{I}\times 100\%$$

where *Q* and *A* are the flow of water across the membrane and the surface area of the membrane, with units of L/h and m^2^, respectively; *C_e_* and *C_i_* refer to the concentrations of effluent and influent, mg/L; *I* represents the total water flow, L/(h), i.e., the flow sum of fresh water across the membrane and the retentate.

### 2.4. Cleaning and Recycling of UF Membranes

Based on the water quality of the influent water, immersion cleaning and chemical cleaning were selected to recover the UF membranes. Various chemical washing methods were compared, including acid immersion using 2% HCl for 30 min, alkaline immersion using 2% NaOH for 30 min, acid/alkaline immersion in succession (2% HCl for 30 min and then 2% NaOH for 30 min), and oxidant/acid immersion in succession (1% H_2_O_2_ for 30 min and then 2% HCl for 30 min). Ultrapure water cleaning for 10 min was applied after each immersion step. The cleaning effect was assessed by the membrane flux (*J*) recovery rate (*η*), i.e., the flow rate of water permeating through a unit membrane module per unit of time [28].

(4) $$\eta =\frac{J_{c}}{J_{0}}$$

where *J_0_* refers to the initial membrane flux of the new UF membrane; and *J_c_* is the membrane flux after cleaning.

### 2.5. Analysis Methods

The DOC of water samples was determined by a Torch total organic carbon (TOC) analyzer (Teledyne Tekmar, Mason, OH, USA) after filtration by cellulose acetate membranes (0.45 µm). BDOC was determined based on a reference method, according to the static cultivation method [29]. The organic molecular weight distribution analysis was conducted using a dead-end UF cell. Membranes with different MW cut-offs were selected to reject organics with various MWs, and then these retained organics were tested by the TOC Analyzer, and the details are described in the Supplementary Materials. The membrane surface morphology of the sample was characterized using a scanning electron microscope (SEM, Hitachi S-3400N, Tokyo, Japan).

## 3. Results and Discussion

### 3.1. DOC and BDOC Removal by Conventional Treatment Processes

**Figure 2:**
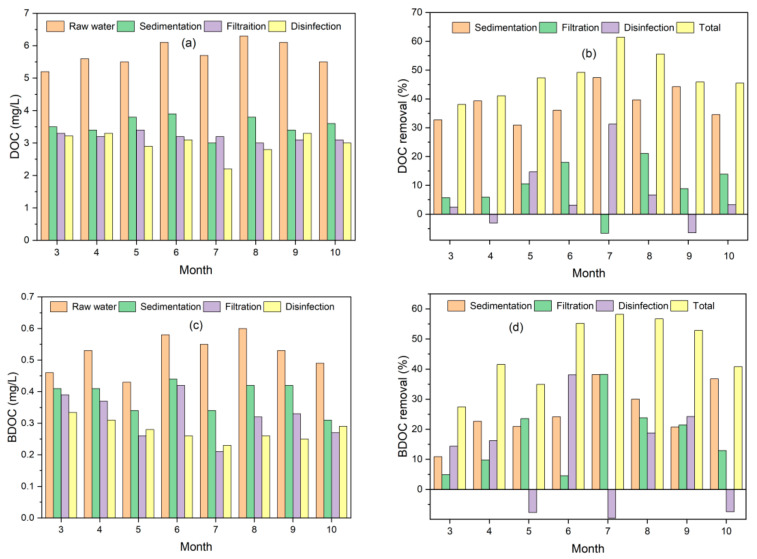
DOC and BDOC removal in the water treatment plant in different months. (**a**) DOC concentrations; (**b**) DOC removals; (**c**) BDOC concentrations; (**d**) BDOC removals.

The DOC and BDOC concentrations in the raw Yangtze River water were determined ranging from 5.2 to 6.3 mg/L and 0.43 to 0.60 mg/L during the investigation, respectively (shown in Figure 2). The DOC and BDOC concentrations were in a relatively stable range, although the concentrations were slightly higher in summer. The DOC concentrations were slightly higher than those in the source region of the Yangtze River in the Tibetan Plateau (1.9–2.4 mg/L) [30], while they were close to the results reported by Zhang et al. [31], with an average value of 5.65 mg/L. The slightly polluted organic matter in water would provide nutrient substrates for microorganisms and also a potential positive relation with antibiotics [32], leading to drinking water bio-instability. Figure S2 shows the molecular weight (MW) distribution of DOC and BDOC in the raw river water, with similar distribution patterns of DOC and BDOC observed. As seen from Figure S2a,b, molecules with MW > 30 kDa of DOC and BDOC were averaged at 27.6 and 24.5%, and those with MW < 1 kDa were 40.3 and 50.8%, respectively, almost half of the total. 

**Figure 3:**
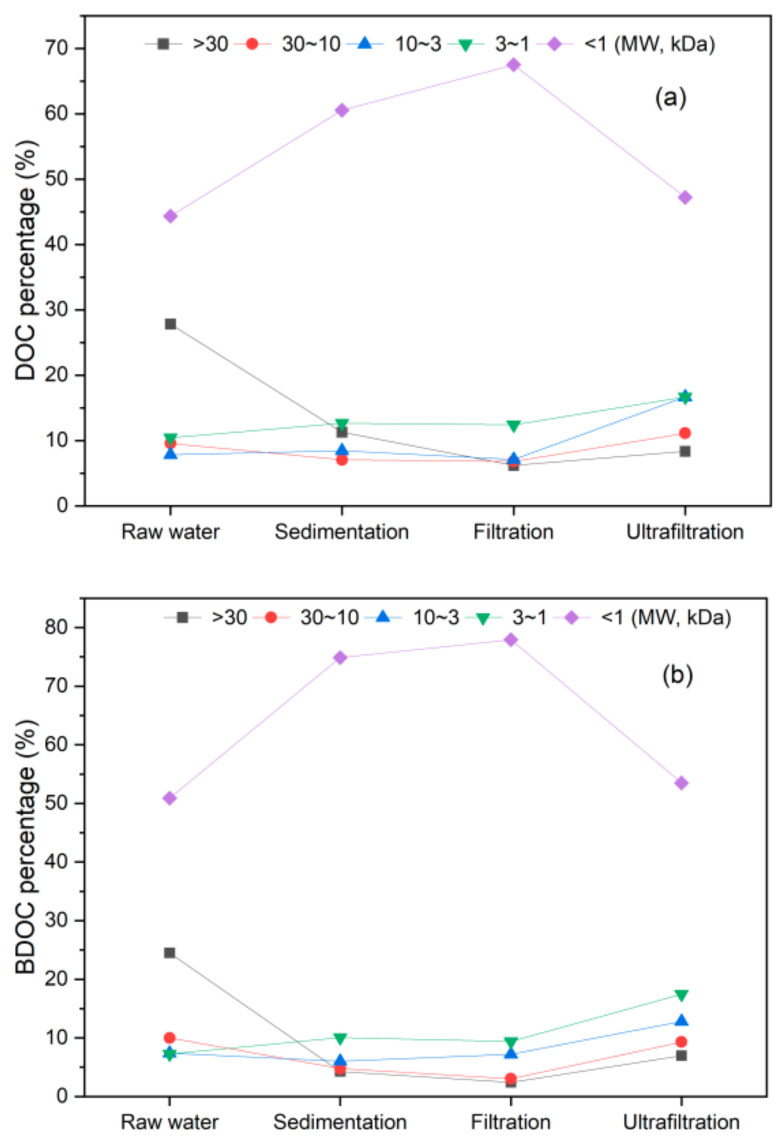
MW distribution of DOC and BDOC in waterworks. (**a**) DOC; (**b**) BDOC.

Figure 2 shows the removal of DOC and BDOC in conventional water treatment processes. The removal of DOC and BDOC in the water plant ranged from 38.0–61.0% and 27.4–58.1%, with residual concentrations of at least 2.3 and 0.22 mg/L, respectively. With these high DOC and BDOC concentrations, the biological stability and safety of the drinking water supply are difficult to guarantee [33]. Limited removal of DOC was also observed during the conventional water treatment processes (coagulation, sedimentation, and sand filtration) in a previous research [34]. The coagulation and sedimentation showed the highest elimination of DOC during treatment processes, with the removal of 30–47%. Sand filtration was an unstable treatment for DOC (−6.6–21%), and disinfection showed only a slight influence. Brooks et al. [35] also reported higher removals of DOC at waterworks where coagulation–flocculation was applied. The possible explanation of the higher removal during coagulation and sedimentation is that dissolved organics with high MW (e.g., >30 kDa and 10–30 kDa) were removed during the process, as the percentage of DOC with MW > 30 kDa decreased and DOC with MW < 1 kDa increased obviously after coagulation and precipitation (shown in Figure 3). Coagulation and sedimentation also caused the highest removal of BDOC, with average removal of 25.5%, lower than that of DOC. This is due to the higher percentage of small MW of BDOC. In addition, from Figure 2b,d, both DOC and BDOC showed higher removal during summer than in other months. Previous studies [36,37] indicate that higher temperatures generally lead to higher coagulation efficiency in organic removal. Moreover, the removal of low-MW organic substances was found to be more difficult at lower temperatures.

Figure 3 shows the percentage change in different MW species in DOC and BDOC during various treatment processes. Organics with different MWs in the water samples were separated using membranes with MW cut-offs of 1, 3, 10, and 30 kDa. The decline in both DOC and BDOC with MW > 30 kDa was observed after sedimentation and sand filtration; while DOC and BDOC with MW < 1 kDa showed opposite trends, because of the removal of those with large MW, indicating the limited removal of both DOC and BDOC with small MW during conventional water treatment processes. The coagulants in the conventional treatment process are easy to associate with macromolecular organic matter with strong hydrophobicity, resulting in electrical neutralization and adsorption bridging, which can destabilize and form large flocs [38]. For the hydrophilic organic matter with smaller MW, it is not easy to combine it with coagulants or for it to be adsorbed by the flocs. Therefore, the conventional processes need to be enhanced to further remove BDOC.

### 3.2. Micro-Polluted Water Treatment by UF

#### 3.2.1. DOC Removal by UF

**Figure 4:**
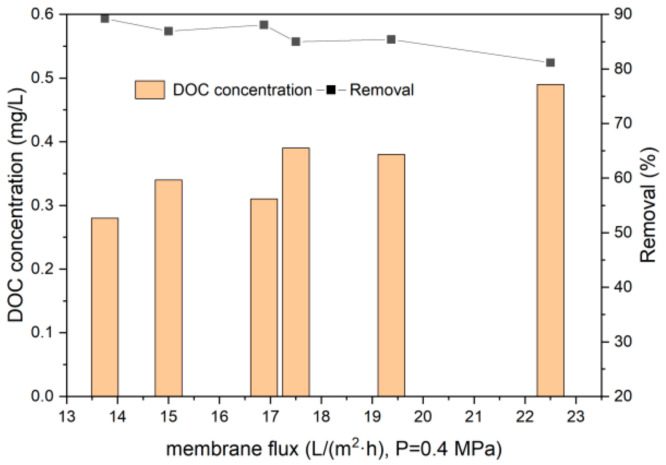
DOC removal from micro-polluted water using UF.

Treatment of micro-polluted water by UF under 0.4 MPa is shown in Figure 4. Under stable pressure, the removal of DOC decreased with the increase in the membrane flux. From Figure 4, the lowest DOC concentration of 0.28 mg/L was observed with a removal of 90%, as the membrane flux of 14 L/m^2^·h was maintained. Previous research showed PES UF membranes (6 kDa) rejected approximately 70% DOC in the feed water, leaving approximately 1.2 mg/L residual DOC [39]. Lee et al. [40] reported 82.7–85.9% removal of DOC by a polyamide UF process (8 kDa). Polyethylene glycol UF membranes (1 kDa) showed above 85% rejection of DOC with an initial concentration of 3.4 mg/L [41], with similar DOC removal as this study. From Figure 3a, the percentage of DOC with MW smaller than 1 kDa decreased obviously after the UF process, indicating the better removal effect of organics with low MW than traditional processes. The fact that the membrane rejects organics with MW larger than 1 kDa is apparent. Yet, a decrease in organics with MW lower than 1 kDa was also observed. 

#### 3.2.2. BDOC Removal by UF

**Figure 5:**
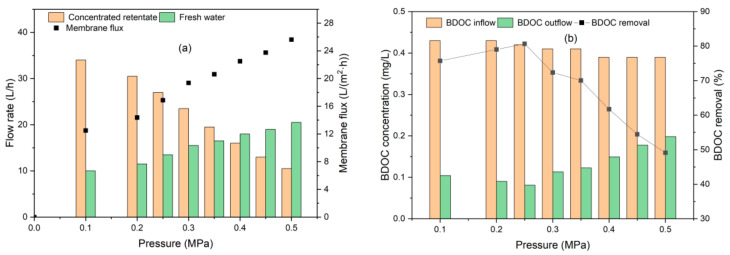
Effect of UF operating pressure. (**a**) Membrane flux; (**b**) removal of BDOC.

Both the retained concentrated solution and permeated freshwater flew back to the tank, keeping a constant BDOC concentration of influent water, to investigate the influence of UF operating pressure. Figure 5 presents the effects of operating pressure on membrane flux, freshwater yield, and BDOC removal under room temperature (25–30 °C) conditions, running for 60 min.

The pressure difference (Δ*p*) on both sides of the UF membrane, i.e., the difference between the average pressure on the water inlet side and the average pressure on the water production side, affected the membrane flux. Since the resistance along the membrane is equal, the pressure drop distribution is uniform. The water production side of the UF module is exposed to the air, so the relative pressure can be regarded as zero. Hence, the pressure difference Δ*p* can be calculated with Equation (5),

(5) $$\Delta p=\frac{p_{1}+p_{2}}{2}-p_{0}$$

where *p*_1_ and *p*_2_ refer to the pressures on the front and end of the membrane inlet side. *p*_0_ is the pressure on the freshwater-producing side, equal to 0.

The membrane separation processes such as ultrafiltration and nanofiltration that use the membrane surface pressure difference as the driving force generally have the following linear relationship without intercepts for pure water [42].

(6) $$J_{w}=\frac{P_{w}}{\delta_{m}}\times \Delta p$$

where *P_w_* is the permeability of the membrane to water, cm^2^/(Pa·s), and it is a constant for pure water, but changes with resistance due to the solute [43]; *δ_m_* refers to the membrane thickness, cm. 

From Figure 5a, with other conditions constant, the UF membrane flux was observed to be proportional to the operating pressure. The membrane flux (*J_w_*) increased with increasing operating pressure (Δ*p*), with a relation of $J_{w}=57.46\times \Delta p\;\left(R^{2}=0.836\right)$. The relatively low R^2^ value indicates a non-perfect linear agreement between *J_w_* and Δ*p* through the coordinate origin because *P_w_* was not a constant during the experiment with the solute resistance increase. The permeate flux of a membrane under little or no fouling observed is different from that exceeding a critical value where rapidly increasing transmembrane pressure and fouling occur [44,45]. With the increase in membrane flux, the contaminants retained by the membrane increased, and the retained organic matter accumulated on the membrane surface, leading to increased hydraulic resistance. To keep the constant flux, the required membrane pressure increases, i.e., the required energy consumption increases. Therefore, the optimal operating pressure should be selected to reduce energy consumption on the premise of ensuring the maximum interception amount. Freshwater yield is a key property of UF membrane efficiency. From Figure 5a, as the operating pressure changed, the water flow through the UF membrane also changed, influencing the freshwater yield correspondingly. As the pressure increased from 0.1 to 0.5 MPa, the freshwater yield enhanced from 22.7 to 66.1%, with the same trend of increased membrane flux.

From Figure 5b, the removal of BDOC via UF increased as the pressure increased from 0.1 to 0.25 MPa, reaching the optimum removal of 80.7%, and then decreased to 51.1% as the pressure continuously increased to 0.5 MPa. The transfer of organics in the influent during the UF process was determined by convection transfer caused by the pressure difference on both sides of the membrane, as well as reverse diffusion, influenced by the concentration gradient. As the pressure increased until concentration polarization occurred, the reverse diffusion of organics was promoted, causing the decline in BDOC removal. The optimal operating pressure of 0.25 MPa was selected, and the highest BDOC removal of 80.7% was observed with the effluent BDOC concentration of 0.08 mg/L, which was much lower than the suggested threshold values of 0.2 mg/L [12] and 0.16 mg/L [46].

**Figure 6:**
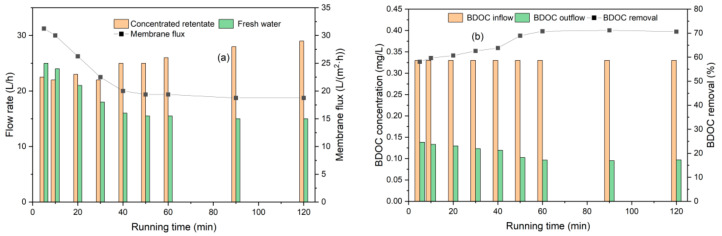
UF performance with time. (**a**) Membrane flux; (**b**) BDOC removal.

The membrane flux and BDOC change with time under a constant pressure of 0.25 MPa, as shown in Figure 6. A decrease in membrane flux (as well as freshwater yield) and an increase in BDOC removal were observed. The decrease in flux during the first 40 min was obvious and then remained stable until 120 min. Furthermore, the BDOC removal increased gradually until 60 min. The initial large flux and low BDOC removal were caused by renewing the slack membrane, and with the rejection and accumulation of pollutants, the flux decreased.

**Figure 7:**
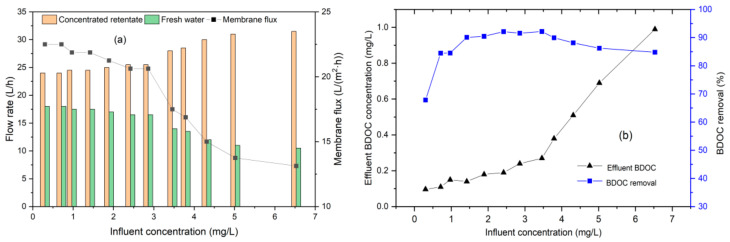
The influence of influent concentration of BDOC on UF performance. (**a**) Membrane flux; (**b**) BDOC removal.

To investigate the effect of influent concentration on BDOC removal, the concentrated water returned back to the tank, and the influent concentration of BDOC increased with the operating time. Room temperature was controlled at 25–30 °C, and the UF process was operated with a constant pressure of 0.25 MPa for 60 min. The impact of the influent concentration on the UF process and BDOC removal is shown in Figure 7.

As the UF process proceeded, the BDOC influent concentration increased from 0.3 to 6.53 mg/L, and membrane flux decreased accordingly. From the data in Figure 7a, the freshwater yields also declined from 42.9 to 25.0%. When the BDOC concentration increased to 3.46 mg/L, the membrane flux suddenly decreased from approximately 21 to 17.5 L/(m^2^·h). Possible reasons include the decreased permeability of solvents and the fouling of the membrane surface after long-term running. From Figure 7b, the effluent BDOC concentration increased with UF running, while the removal of BDOC showed a trend of increasing first and then slightly decreasing when the BDOC concentration exceeded 3.2 mg/L. Removal was maintained at higher than 90% for influent BDOC concentrations ranging from 1.5 to 4.0 mg/L. However, when the influent BDOC concentration was higher than 2.4 mg/L, the effluent concentration exceeded 0.2 mg/L, which is the suggested limit of the biostability of water. 

### 3.3. UF Membrane Fouling and Cleaning

**Figure 8:**
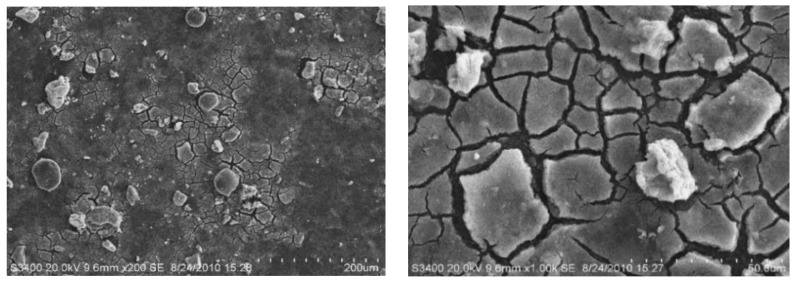
SEM photos of used UF membrane.

The SEM photos of pre-micro-filter can be seen in Figure S3, and Figure 8 shows the polluted UF membrane surface. It was found that the membrane pores on the inner surface of the membrane were almost completely covered by a thick layer of pollutants and were invisible. Fouling types of UF membranes applied for surface-water treatment include organic, inorganic, and microbial [28]. Massive scaling on the surface was clearly observed. The surface of the pollutants was rough, and microbial cells were attached. Due to the long-term operation of the membrane modules, the membrane fouling issue was mainly caused by the adsorption of solutes, the precipitation of particulate matter, and some biological matters. During the operation of UF, a gel layer was formed on the surface of the membrane due to the solute concentration around the membrane reaching its solubility limit and solute precipitating onto the membrane surface. Without removing the accumulated pollutants and reducing the membrane fouling, the membrane flux would be seriously affected, thereby increasing the operating energy and decreasing the treatment efficiency.

**Figure 9:**
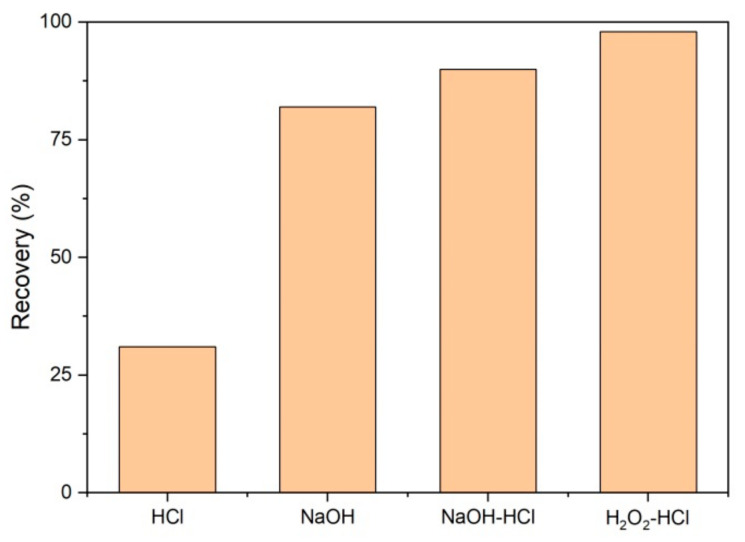
The cleaning effects of UF membranes.

Figure 9 shows the cleaning effect of fouling UF membranes. When washed with 2% hydrochloric acid, the membrane flux was restored to 31% of that before fouling; and when the UF membrane was washed with a 2% sodium hydroxide solution, the membrane flux was restored to 82%, much higher than when using acid. When combining alkali washing and acid washing in turn, the membrane flux was recovered up to 90%. A slight increase in removal was observed at 8% compared with only using alkali. Similarly, Razavi et al. [47] reported rinsing to be ineffective at removing membrane fouling using an acid solution, and caustic cleaning was more effective. This could be mainly related to the adsorption and deposition of various types of pollutants on the membrane surface. Inorganic scales, precipitates, and metal oxides can be dissolved in an acid solution, but their decomposition is difficult in a high-pH solution [21,28]. In contrast, alkali can promote the rapid hydrolysis of organic matter, e.g., proteins and polysaccharides can be decomposed to small amides and sugars [48,49]. Alkali can also decrease the number of bonds between the foulant and the membrane surface, and then increase the mass transfer of cleaning chemicals to the surface. Therefore, when washed with alkali before acid, the organic matter on the surface of the membrane is quickly removed by the alkali solution, increasing the mass transfer of acid, and the inorganic scale can be then dissolved by acid effectively. Compared with only alkaline washing, the effect of alkaline washing–acid washing on membrane cleaning was not significantly improved, indicating membrane fouling was mainly caused by organic pollutants. The recovery rate of membrane flux after H_2_O_2_ washing–acid washing was 98%, better than that of alkali washing–acid washing. The possible reason for the higher membrane clean efficiency is that the oxidizing substances (H_2_O_2_) oxidize and decrease the organic pollutants deposited on the surface of the membrane rapidly, and also eliminate microbes and their secretions effectively [50], so that acid can then react with inorganic scales directly without the influence of organic matter.

## 4. Conclusions

Biodegradable dissolved organic carbon (BDOC) in river water sources affects the drinking water quality and safety in the urban water supply system, as conventional treatment processes of water plants generally find it difficult to reduce them to meet the requirements of biological stability. The molecular weight (MW) of organics in the water source of the Yangtze River was observed to be mainly distributed in a range of higher than 30 kDa and lower than 1 kDa. The traditional technology of “coagulation-sedimentation-filtration-disinfection” in waterworks removed dissolved organic carbon (DOC) effectively with MW > 30 kDa from Yangtze River water, but the BDOC elimination only ranged between 27.4 and 58.1%, due to their predominant MW being smaller (<1kDa). Coagulation and sedimentation showed the highest removal of organics among conventional processes. A pilot-scale ultrafiltration process (UF, aromatic polyamide roll membrane with a molecular weight cut-off of 1 kDa) was operated in this study after sand filtration as an advanced treatment to remove BDOC. UF membrane flux decreased with the increase in the influent BDOC concentration and the decrease in the operating pressure. With the optimal operating pressure of 0.25 MPa, the removal of BDOC by UF could reach 80.7%, leading to the lowest effluent BDOC concentration of 0.08 mg/L. To ensure BDOC concentrations after UF are always below 0.2 mg/L, we suggest controlling the influent BDOC to be lower than 2.4 mg/L. The fouling of the membrane was observed through SEM. Membrane fouling was caused by organic, inorganic, and microbial matter. Alkaline washing showed a better effect than acid washing while the H_2_O_2_–HCl washing method had the optimal cleaning effect on the membrane surface, with 98% recovery of the membrane flux.

## Data Availability

The data presented in this study are available on request from the corresponding author.

## Supplementary Materials

The following supporting information can be downloaded at: https://www.mdpi.com/article/10.3390/polym14214689/s1, Figure S1: Molecular weight analysis of organics; Figure S2: MW distribution of DOC (a) and BDOC (b) of the lower reaches of Yangtze River; Figure S3: SEM photos of new microfiltration membranes (a and b) and fouling microfiltration membranes (c and d); Table S1: The water sample quality for UF process.
