# Supplementary material for: Biodegradable Dissolved Organic Carbon (BDOC) Removal from Micro-Polluted Water Source Using Ultrafiltration: Comparison with Conventional Processes, Operation Conditions and Membrane Fouling Control
Source: Polymers (Basel). 2022 Nov 3;14(21):4689. doi: 10.3390/polym14214689 (PMC9658970; doi:10.3390/polym14214689)
Supplement: Supplementary file 1 [file polymers-14-04689-s001.zip › polymers-1964577-supplementary.pdf]

Supplementary material

Biodegradable Dissolved Organic Carbon (BDOC) Removal from Micro-Polluted Water Source Using Ultrafiltration: Comparison with Conventional Processes, Operation Conditions and Membrane Fouling Control

Ming Chen [1,*], Shuhuai Shen [1], Fan Zhang [2], Cong Zhang [1] and Jianglei Xiong [3,*]

1 School of Civil Engineering, Southeast University, Nanjing 210096, China
2 Huzhou Ecological Environment Bureau, Changxing Branch, Huzhou 313100, China
3 China Electronics System Engineering No.2 Construction Co. Ltd, Wuxi 214115, China
* Correspondence: chenm@seu.edu.cn (M.C.); xiongjianglei@cese2.com (J.X.)

Molecular weight analysis of organics

Organics with different molecular weight (MW) were separated using a dead-end UF cell, shown in **Figure S1**. The photo shows the dead-end UF cell and the diagram indicates the analysis steps. The volume of the cell was 350 mL with an effective filtration area of 3.32×10^{-3} m^2. Nitrogen gas was used as driven force to give the cell a pressure ranged from 0.1 to 0.3 MPa. UF membranes with different MW cut-offs were selected to reject organics with various MWs, and then these retained organics were tested by total organic carbon (TOC) analyzer. Membranes were purchased from MILLIPOR, with MW cut-offs of 1, 3, 10, and 30 kDa.

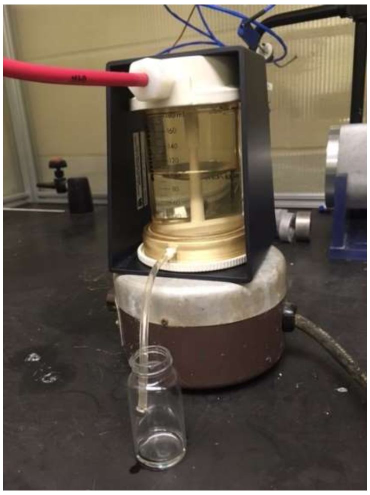

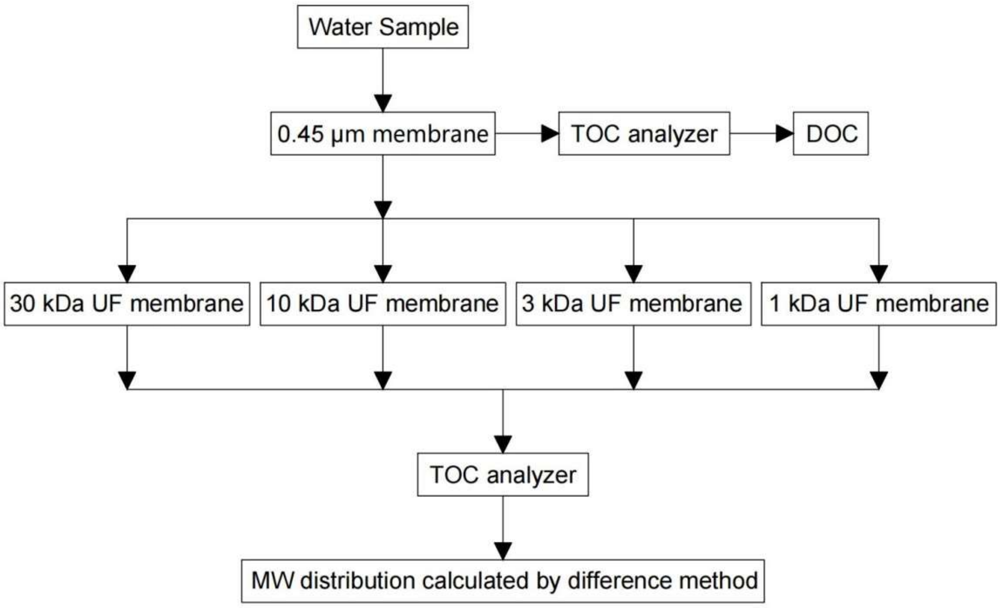

Figure S1. Molecular weight analysis of organics.

Table S1. The water sample quality for UF process

	COD_{Mn} (mg/L)	UV_{254} (cm^{-1})	Turbidity (NTU)	DOC (mg/L)	BDOC (mg/L)
Maximum	2.2	0.087	0.25	3.2	0.34
Minimum	1.8	0.061	0.10	2.0	0.30
Average	1.95	0.073	0.15	2.6	0.318

COD_{Mn} was determined using the standard method, and UV_{254} was measured using UV-Vis spectrophotometer (Spectrum Instruments, Shanghai). Turbidity was determined by Hach 2100P portable turbidity meter.

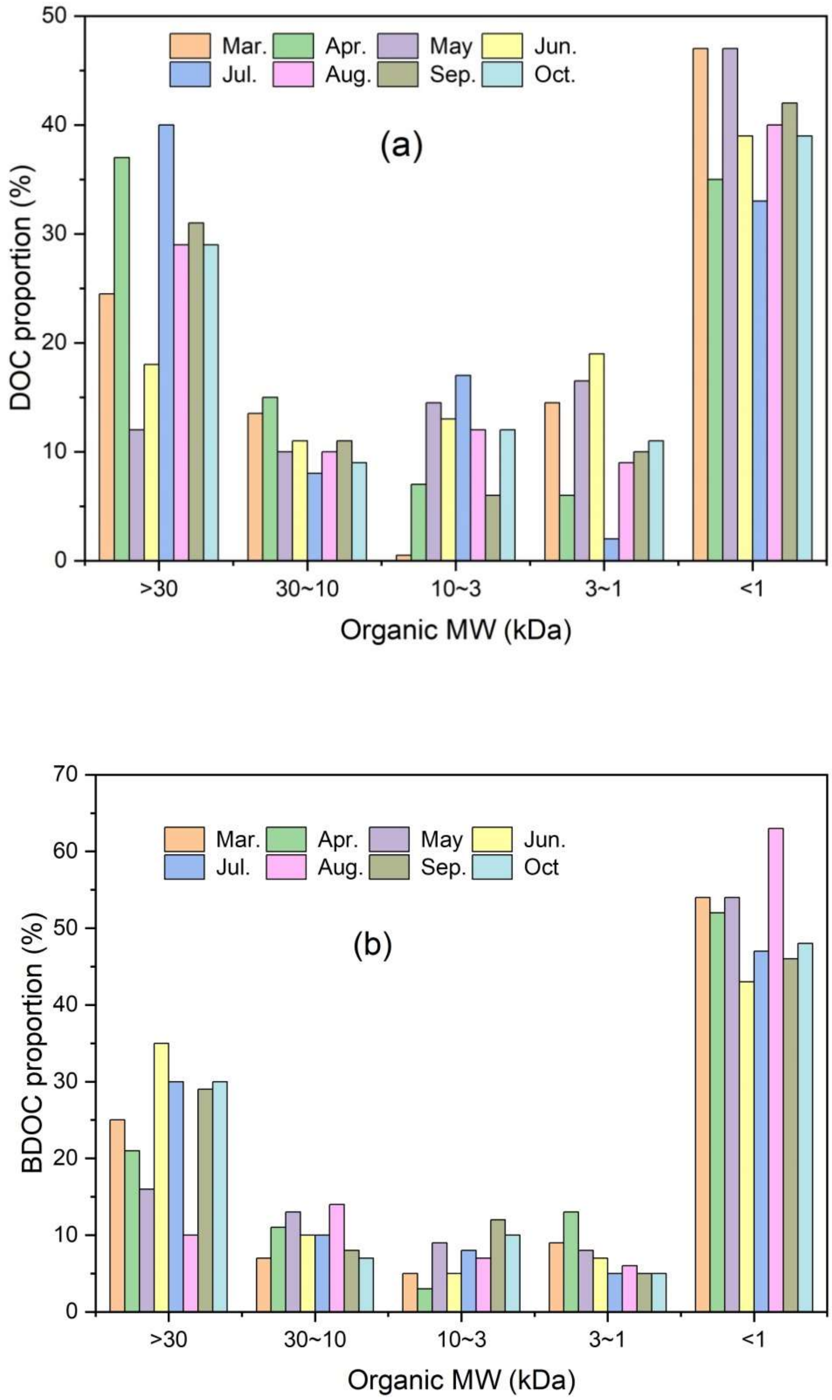

Figure S2. MW distribution of DOC (a) and BDOC (b) of the lower reaches of Yangtze River.

Figures S2a and **S2b** show the MW distribution of DOC and BDOC of Yangtze River in various months. Molecules with MW > 30 kDa of DOC and BDOC were 27.6 and 24.5%, and those with MW < 1 kDa were 40.3 and 50.8% respectively.

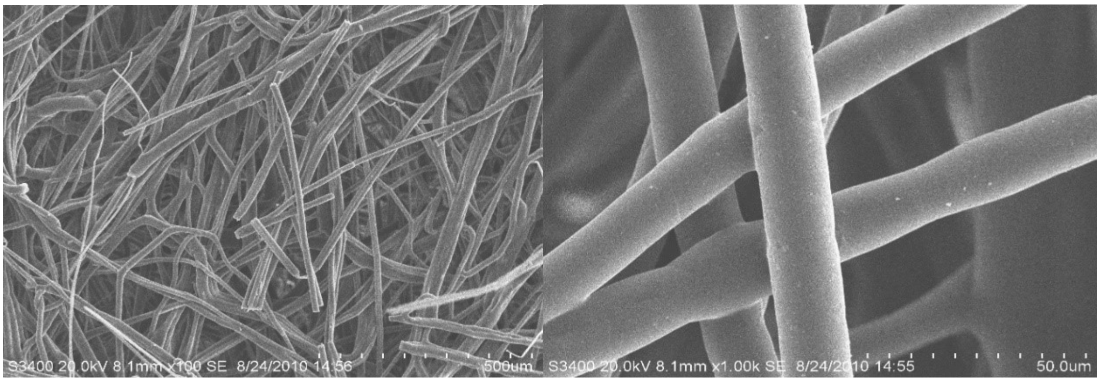

(a) new membrane (100 times）   (b) new membrane 1000 times

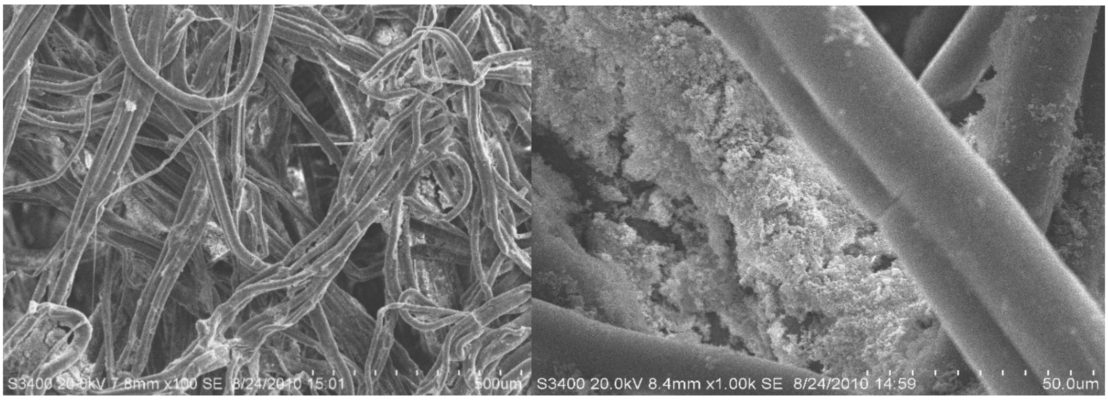

(c) polluted membrane (100 times)   (d) polluted membrane (1000 times)

Figure S3. SEM photos of new microfiltration membranes (a and b) and fouling microfiltration membranes (c and d).
